# Localization of Epileptogenic Zone Based on Cortico-Cortical Evoked Potential (CCEP): A Feature Extraction and Graph Theory Approach

**DOI:** 10.3389/fninf.2019.00031

**Published:** 2019-04-24

**Authors:** Cui Zhao, Ying Liang, Chunlin Li, Runshi Gao, Jing Wei, Rui Zuo, Yihua Zhong, Zhaohui Ren, Xinling Geng, Guojun Zhang, Xu Zhang

**Affiliations:** ^1^Beijing Key Laboratory of Fundamental Research on Biomechanics in Clinical Application, Capital Medical University, Beijing, China; ^2^School of Biomedical Engineering, Capital Medical University, Beijing, China; ^3^Department of Functional Neurosurgery, Xuanwu Hospital, Capital Medical University, Beijing, China

**Keywords:** epilepsy, CCEP, ECoG, effective connection, graph theory

## Abstract

**Objective:**

Epilepsy is a chronic brain disease, which is prone to relapse and affects individuals of all ages worldwide, particularly the very young and elderly. Up to one-third of these patients are medically intractable and require resection surgery. However, the outcomes of epilepsy surgery rely upon the clear identification of epileptogenic zone (EZ). The combination of cortico-cortical evoked potential (CCEP) and electrocorticography (ECoG) provides an opportunity to observe the connectivity of human brain network and more comprehensive information that may help the clinicians localize the epileptogenic focus more precisely. However, there is no standard analysis method in the clinical application of CCEPs, especially for the quantitative analysis of abnormal connectivity of epileptic networks. The aim of this paper was to present an approach on the batch processing of CCEPs and provide information relating to the localization of EZ for clinical study.

**Methods:**

Eight medically intractable epilepsy patients were included in this study. Each patient was implanted with subdural grid electrodes and electrical stimulations were applied directly to their cortex to induce CCEPs. After signal preprocessing, we constructed three effective brain networks at different spatial scales for each patient, regarding the amplitudes of CCEPs as the connection weights. Graph theory was then applied to analyze the brain network topology of epileptic patients, and the topological metrics of EZ and non-EZ (NEZ) were compared.

**Results:**

The effective connectivity network reconstructed from CCEPs was asymmetric, both the number and the amplitudes of effective CCEPs decreased with increasing distance between stimulating and recording sites. Besides, the distribution of CCEP responses was associated with the locations of EZ which tended to have higher degree centrality (DC) and nodal shortest path length (NLP) than NEZ.

**Conclusion:**

Our results indicated that the brain networks of epileptics were asymmetric and mainly composed of short-distance connections. The DC and NLP were highly consistent to the distribution of the EZ, and these topological parameters have great potential to be readily applied to the clinical localization of the EZ.

## Introduction

Epilepsy is one of the most common and chronic neurological disorders and is usually caused by excessive and abnormal firing of neurons in the brain cortex. Epilepsy is characterized by recurrent seizures and the symptoms can be diverse, including staring, tonic movements, muscle spasms and impaired consciousness (Pitkänen et al., 2016). The pathogenesis of epilepsy is complex as a result of the multifactorial nature and its heterogeneity. For example, brain injury, stroke and genetic mutations, can all induce epilepsy (van Mierlo et al., 2014). Globally, approximately 70 million people have epilepsy, and up to 30% of these patients have medically intractable epilepsy (Singh and Trevick, 2016). In China, 9 million people suffer from epilepsy, a condition which is usually treated with anti-epileptic drugs (AEDs). While the effects of AEDs are not satisfactory, one possible option is resective surgery of the epileptogenic zone (EZ), a procedure which can benefit patients by reducing or eliminating seizure activity (Vos et al., 2016; Yue et al., 2017). However, incomplete resection of the focus, or damage incurred by normal brain regions during surgery may fail to achieve an effect, or may even aggravate the condition (van Mierlo et al., 2014). Precise delineation of the EZ is the key to epilepsy surgery; however, abnormal connectivity of epileptic networks makes it difficult for the clinicians to delineate the epileptogenic focus unambiguously.

Electroencephalography (EEG) is one of the most important techniques for the diagnosis and treatment of epilepsy patients. EEG can record the electric signals generated by neurons in the brain with higher temporal resolution than magnetic resonance imaging (MRI), positron emission tomography (PET) and other techniques, and is also easy to operate, which can reveal the neural mechanism of human brain during complex cognitive and affective tasks and contribute unique information for the advance of neuroscience (Yan et al., 2017a,b). EEG is now universally regarded as the gold standard for the localization of EZ. Electrocorticography (ECoG) uses electrodes implanted on the surface of the cortex, which can provide recording and stimulation data directly from the cortical surface of the human brain. With high temporal resolution, good spatial resolution and high signal-noise ratio, ECoG has been widely used in preoperative assessment for resection surgery (Enatsu and Mikuni, 2016). Matsumoto et al. (2004) were the first to use low-frequency electrical stimulation to the cortex in eight refractory epilepsy patients, and analyzed the distribution of response potentials to study the connectivity of the language network; this method was termed cortico-cortical evoked potential (CCEP). CCEP is the response potential recorded at one cortical region when a single pulse of electrical current was applied at another remote location of the cortex. This technique allows us to evaluate effective connectivity between the stimulating and recording sites or in different cortical regions, thus providing information on the direction of connectivity, which cannot be detected by functional magnetic resonance imaging (fMRI), diffusion tensor imaging (DTI) or any other imaging methods (Koubeissi et al., 2012).

Furthermore, epilepsy is a complex network disease associated with spatial organization of epileptic cortices, functional connectivity alternations and pattern of seizure, the abnormal connectivity of epileptic network makes it difficult to localize the EZ (Mears and Pollard, 2016). In recent years, the application of CCEP and the advancements of other neuroimaging techniques have brought about great progress in the precise localization of the EZ and human brain network mapping (Araki et al., 2015; Kamada et al., 2017; Fox et al., 2018). It is also important to mention that graph theory provides significant benefit for the studies of brain network connectivity, which is now widely used to analyze data arising from EEG, MRI, and fMRI (Sha et al., 2017; Yan et al., 2018). As a method of network analysis, graph theory is the study of graphs, which are mathematical structures used to model pairwise relations between objects. A graph is made up with nodes, which are connected by edges (Bullmore and Bassett, 2011). Analyzing CCEP with graph theory can provide meaningful descriptions of large-scale brain networks, and this method has been shown to provide a means to probe the human brain network and to evaluate the cortical excitability (Vecchio et al., 2015; Keller et al., 2018; Parker et al., 2018).

Due to the huge amounts of data created by EEG, the complexities of data processing and the lack of a systematic method for reconstructing the brain network based on CCEPs, there are still some difficulties in the clinical application of CCEPs. In this study, we used CCEP mapping in a cohort of refractory epilepsy patients implanted with ECoG electrodes, and measured the topological properties of the brain network by graph theory in order to offer a convenient and effective batch processing application of CCEPs and help the clinicians localize the EZ in a precise manner.

## Methods

### Subjects

Eight subjects (7 males and 1 female; mean age: 21.5 years, range: 13–28 years) with medically intractable epilepsy were enrolled at Beijing Institute of Functional Neurosurgery at Xuanwu Hospital Capital Medical University. All patients were implanted with subdural grid electrodes for the invasive evaluation for epilepsy surgery. Patients’ demographic characteristics and clinical information are illustrated in Table 1. The EZ was defined by experienced clinical epileptologists with comprehensive based on the resected areas in epilepsy surgery, combing with the postoperative pathology results, long-term video EEG recordings, clinical symptoms and neuroimaging. The other implanted brain area out of EZ was defined as non-EZ (NEZ). The prognoses of all patients involved in this study are overall good. All patients involved in this study gave their informed consent and all procedures were approved by the Medical Research Ethics Committee at Xuan Wu Hospital of Capital Medical University.

**Table 1:** Clinical information of the patients.

Patient	Gender/Age	Epileptic foci	Implanted side	Number of electrodes	Invested lobes
P1	M/20	R Temporal	R	96	Frontal, Parietal, Temporal
P2	M/23	L Temporal	L	96	Frontal, Parietal, Temporal
P3	M/26	R Temporal	R	96	Frontal, Parietal, Temporal
P4	F/28	L Parietal	L	112	Frontal, Parietal, Temporal
P5	M/23	L Parietal	L	64	Frontal, Parietal, Temporal, Occipital
P6	M/16	L Parietal, Postcentral gyrus	L	80	Frontal, Parietal, Temporal,
P7	M/23	L Parietal	L	64	Parietal, Occipital, Precentral gyrus, Postcentral gyrus
P8	M/13	L Parietal, Occipital	L	104	Frontal, Parietal, Temporal, Occipital

*M, male, F, female; R, right and L, left.*

### CCEP Procedure

During the pre-surgical evaluation, single-pulse stimulations were delivered to pairs of adjacent electrodes with a bipolar setup. Stimulation was conducted with a constant current square wave pulse which was 0.3 ms in duration, a pulse frequency of 1 Hz, and 50 trials per electrode pair. ECoG was continuously recorded with a 128-channel digital EEG system at 1024 Hz. Patients were awake and remained still at the time of CCEP recording. All programming was performed in Matlab R2016b (The MathWorks Inc., Natick, MA, United States).

### Preprocessing and Feature Extraction of CCEPs

First, the responses of each channel over the same stimulation electrodes were averaged with a time window of 1000 ms, time-locked to the stimulus (the stimulus was set as zero point, 200 ms pre-stimulation and 800 ms post-stimulation). After averaging, the baseline drift of CCEP on each channel was eliminated, the interval between −100 ms and −5 ms prior to the stimulation pulse was set as baseline (Trebaul et al., 2016). Analyses of CCEP were conducted on electrode-pair level and on region level. The gross anatomy atlas and Brodmann’s Areas (BA) atlas were used to parcellate the brain area implanted with electrodes into several regions. Each electrode was assigned to a specific brain region of the atlas. Original ECoGs were averaged according to paired electrodes or among the same brain regions.

Each CCEP consists of an early sharp negative response (N1, 10–50 ms post-stimulation) and a subsequent slow-wave (N2, 50–300 ms post-stimulation) (Matsumoto et al., 2017). Here we only focused on the earliest response. Combining with the waveform characteristics of CCEP and the characteristics of ECoG signals actually acquired in this research, we decided to set the largest peak of CCEP during the period of 16–40 ms post-stimulus as the index of connectivity between the stimulating and recording sites. The first 16 ms was excluded from our analysis due to stimulation artifacts. In order to reduce the effect of variations among different channels, the amplitudes of CCEP at each site were normalized and converted into Z-scores.

### Effective Network Construction and Graph Theorical Analysis

In this paper, the normalized CCEP amplitudes were set as the connection between two sites (electrode pairs or regions). Three different kinds of weighted connectivity matrices were observed from CCEPs: (1) connected matrices based on electrode-pairs, (2) connected matrices based on gross anatomy atlas, and (3) connected matrices based on BA atlas. Each row corresponding to a stimulation site and each column to a recording site. Then, a threshold was set as six times the standard deviation (SD) to identify the effective CCEP connectivity for each patient (Keller et al., 2014). If the amplitude of CCEP exceeded the threshold, the connectivity from the stimulating site to the recording site was effective, the corresponding element in binary connected matrix was set to value “1,” if not, the connectivity was ineffective, and the corresponding element in binary matrix was set to value “0.” Thus, three kinds of binary matrices were generated for each patient (electrode-pair level, gross anatomy-region level and BA-region level), which were then applied as masks to captured the underlying effective CCEP connectivity in the corresponding CCEP-weighted connectivity matrices. Finally, three different effective CCEP networks were reconstructed.

In order to characterize network topology, graph theory mathematical techniques were employed to analyze CCEP matrices. The electrode pairs and brain regions were defined as nodes of the network, and the effective CCEP amplitudes were defined as the edges (Rubinov and Sporns, 2010; Bullmore and Bassett, 2011). We computed widely used complex network measures to analyze the topological properties of the brain network in a quantitative manner, as detailed below.

Betweenness centrality (BC): a measure of centrality in a network based on shortest paths.

(1) $${\text{BC}}_{i}=\sum_{i\neq j\neq k\in \text{G}}\frac{\delta_{\mathrm{ij}}(k)}{\delta_{\mathrm{ij}}}$$

Where *δ_ij_* is the number of shortest paths between node *i* and *j* within network *G*, and *δ_ij_* (*k*) is the number of those paths which pass through node *k*.

Degree centrality (DC): it reflects the information communication ability of the given node in the network, which is defined as the sum of all neighboring link weights.

(2) $${\text{DC}}_{i}=\sum_{j=1}^{N}a_{\mathrm{ij}}\;(i\neq j)$$

*N* is the total number of nodes in the network *G*, *a_ij_* indicates the effective connection between node *i* and *j*, which is the amplitude of effective CCEP recorded at node *j* when node *i* was stimulated.

Nodal clustering coefficient (NCP): a measure of the degree to which nodes tend to cluster together in the network *G*.

(3) $${\text{NCP}}_{i}=\frac{E_{i}}{\frac{1}{2}k_{i}(k_{i}-1)}$$

Where *E_i_* denotes the number of edges that was actually connected with node *i*, and *k_i_* is the number of neighbors of node *i*. If a node *i* have *k_i_* neighbors, ½*k_i_* (*k_i_* − 1) edges could exist among this node.

Nodal efficiency (NE): it characterizes the efficiency of parallel information transfer of a given node in this network.

(4) $$\text{NE}(i)=\frac{1}{N-1}\cdot \sum_{j,j\neq i}\frac{1}{d_{\mathrm{ij}}}$$

*d_ij_* denotes the length of the shortest path between node *i* and node *j*.

Nodal local efficiency (NLE): a measure of the information exchanged among the immediate neighbor nodes, when node *i* is removed.

(5) $$\text{NLE}(i)=\frac{1}{{\text{k}}_{i}({\text{k}}_{i}-1)}\cdot \sum_{\text{j}\in G_{i}}\frac{\sum_{h\in G_{i}}d_{\mathrm{jh}}}{{\text{k}}_{i}-1}$$

Where *G_i_* is the local subnetwork consisting only of a node *i*’s immediate neighbors, but not the node *i* itself, *k_i_* is the number of nodes in subnetwork *G_i_*.

Nodal shortest path length (NLP): it quantifies the mean distance of routing efficiency between the given nodes *i* and the other nodes in the network.

(6) $${\text{NLP}}_{i}=\frac{1}{N(N-1)}\sum_{i,j,i\neq j}d_{\mathrm{ij}}$$

### Statistical Analysis

We used Pearson’s correlations to assess how effective CCEPs related to the distance between stimulating and recording sites. Additionally, to illustrate the differences between the topological properties of EZ and NEZ, the computed topological properties of electrode-pairs and parcellated regions located in EZ and NEZ were averaged. And paired-sample *t*-test was used to test for group difference of EZ and NEZ in network topologies.

## Results

### Temporal and Spatial Distribution of CCEPs

**FIGURE 1:**
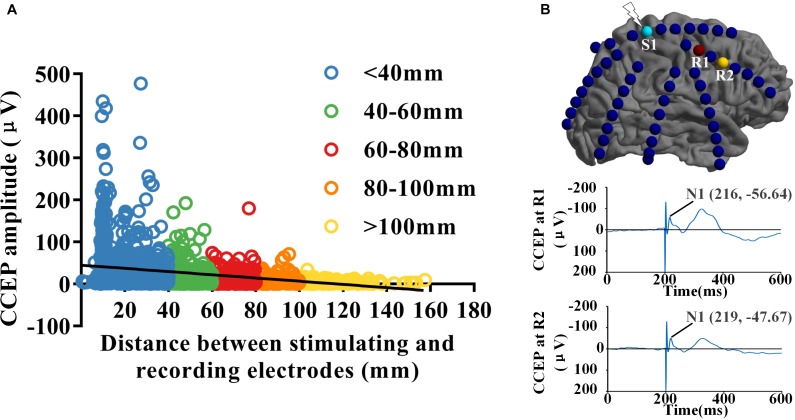
Spatial and temporal distribution of CCEPs. **(A)** The distribution of CCEPs across the distance between stimulating and recording electrode pairs. The effective CCEP responses became less and the amplitudes got lower when the distance increasing. **(B)** When electrode S1 was stimulated, the CCEP responses at different recording sites (R1 and R2) was shown respectively. Potential N1 of CCEP recorded at R1 had higher amplitude and smaller latency than the one at R2, which located farther to S1 than R1.

Eight drug resistant epilepsy patients with different anatomical EZ locations were included in this study, a total of 712 contacts were implanted. Thousands of CCEP responses were recorded with subdural electrode strips when low-frequency electrical stimulus was applied to the cortex directly. We reconstructed three connectivity networks with different spatial scales, electrode-pair-level and region-level based on the gross anatomy and BA atlas. Distance between electrode-pairs was calculated using the Euclidean distance between the midpoints of the electrodes of each pair (Keller et al., 2014). The strength of effective CCEPs decreased significantly with the increase of distance between stimulating and recording sites (*R* = −0.335, *P* < 0.001). As shown in Figure 1A, when the distance increasing, the effective CCEPs became less and the amplitudes became lower. Figure 1B shows the CCEP responses at different recording sites (R1 and R2), when electrode S1 was stimulated. The one (R1) closer to the stimulating site had higher amplitude and smaller latency than the farther one (R2).

### Topologies of EZ and NEZ in Effective Brain Networks

Two different templates were used to parcellate the brain areas of epileptics, the gross anatomy atlas based on Nissl plates and the BAs atlas defined by cytoarchitectural organization of neurons. We constructed two region-level brain networks for each patient. These parcellated regions were classified into two categories, one located in the EZ and the other located in NEZ. We computed the widely used graph theoretical measures to characterize the topological properties of brain networks, including BC, DC, NCP, NE, NLE, and NLP, and compared the topologies of EZ and NEZ.

#### Connectivity Analysis Based on the Gross Anatomy Atlas

**FIGURE 2:**
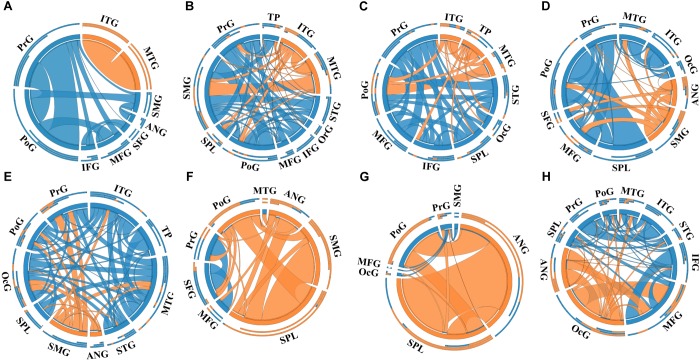
The weighted brain networks reconstructed with the effective CCEP responses, using the gross anatomy atlas to parcellate brain regions. Nodes are represented by circularly arranged segments, of which located in epileptogenic zone (EZ) is colored by orange and the others blue. Edges are presented with ribbons of which connected with EZ regions are colored by orange and the others blue. The stronger the connection is, the thicker the connected ribbon is. Each ribbon has a direction, it starts at the stimulated/outgoing region which it touches, and ends at the recording/ingoing region which it does not touch. The three outer rings are stacked bar plots that represent relative contributions of a region (outgoing/ingoing/totally). Panels **(A–H)** were the weighted brain networks corresponding to patients P1–P8. Abbreviations: PrG, precentral gyrus; PoG, postcentral gyrus; OrG, orbital gyri; SFG, superior frontal gyrus; MFG, middle frontal gyrus; IFG, inferior frontal gyrus; SPL, superior parietal lobule; SMG, supramarginal gyrus; ANG, angular gyrus; STG, superior temporal gyrus; MTG, middle temporal gyrus; ITG, inferior temporal gyrus; TP, temporal pole and OcG, occipital gyrus.

**FIGURE 3:**
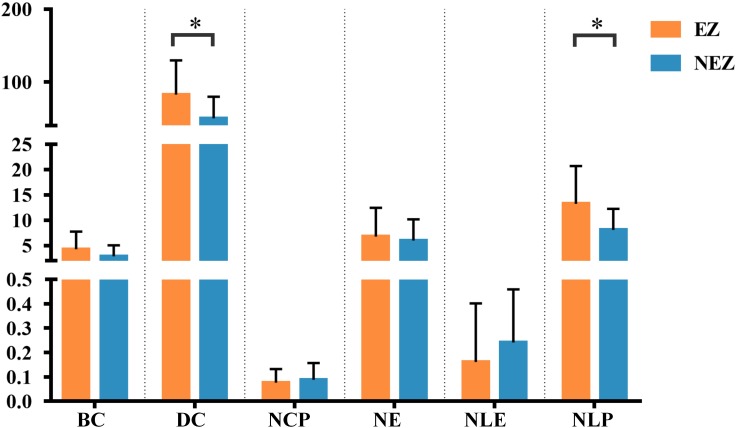
The weighted brain network reconstructed from the CCEP responses based on the BA atlas. Regions located in EZ were colored by orange, and the others in non-epileptogenic zone (NEZ) were colored by blue. Abbreviations: BC, betweenness centrality; DC, degree centrality; NCP, nodal clustering coefficient; NE, nodal efficiency; NLE, nodal local efficiency; NLP, nodal shortest path length. ^∗^*P* < 0.05.

A total of 14 regions of the gross anatomy atlas were involved in this study with a mean of 9 (min–max: 7–11) per patient. The constructed brain networks of patients P1–P8 were shown in Figure 2. Regions located in EZ tended to strongly connected with each other in most of the epileptic patients. When pairs of electrodes in EZ were stimulated, the effective CCEP responses with high amplitudes usually located in regions of EZ. The distributions of the graph metrics averaged across all patients are presented in Figure 3. Significant differences were observed in DC and NLP (paired-sample *t*-test, *P* < 0.05) between EZ and NEZ. Compared with that in NEZ, DC, and NLP significantly increased in EZ, which means that regions in EZ have high integration in the effective brain networks. While, the other graph metrics (BC, NCP, NE, and NLE) did not show any significant difference between EZ and NEZ.

#### Connectivity Analysis Based on the BA Atlas

**FIGURE 4:**
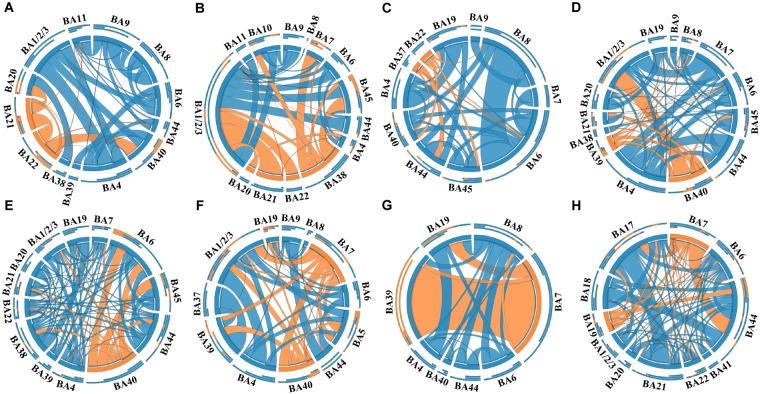
The weighted brain networks reconstructed with the effective CCEP responses, using the Brodmann’s area (BA) atlas to parcellate brain regions. Nodes are represented by circularly arranged segments, of which located in EZ were colored by orange and the others blue. Edges are presented with ribbons of which connected with EZ regions are colored by orange and the others blue. The stronger the connection is, the thicker the connected ribbon is. Each ribbon has a direction, it starts at the stimulated/outgoing region which it touches, and ends at the recording/ingoing region which it does not touch. The three outer rings are stacked bar plots that represent relative contributions of a region (outgoing/ingoing/totally). Panels **(A–H)** were the weighted brain networks corresponding to patients P1–P8.

**FIGURE 5:**
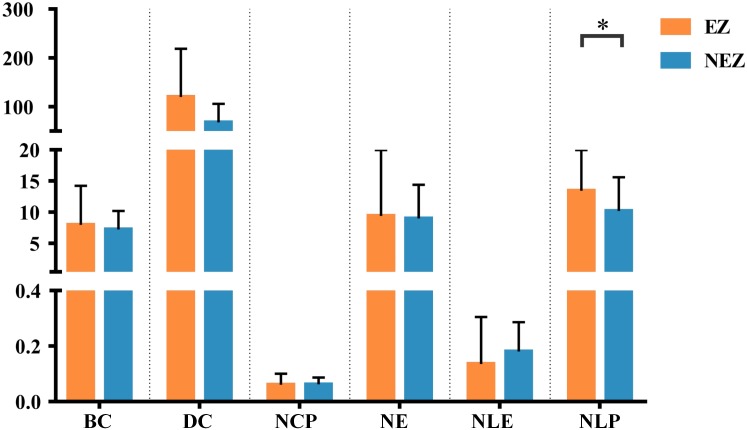
The graph metrics of brain networks reconstructed with the effective CCEP responses, using the BA atlas to parcellate brain region. Regions located in EZ were colored by orange, and the others in NEZ were colored by blue. ^∗^*P* < 0.05.

In this study, a total of 23 regions of BA atlas were used for network construction with a mean of 12 (min–max: 8–13) per patient. Figure 4 presents the weighted brain networks of the eight patients studied in this study. Regions in EZ were also strongly connected with each other in the effective networks base on BA atlas, which is similar to the connectivity of the networks reconstructed with the gross anatomy atlas. As shown in Figure 5, NLP of EZ were significantly higher than that in NEZ (paired-sample *t*-test, *P* < 0.05), which was consistent with the results computed based on the gross anatomy atlas. While DC (paired-sample *t*-test, *P* = 0.081), BC and other topological properties did not show any significant difference between EZ and NEZ in the effective networks reconstructed based on the BA atlas. The insignificant difference of the distribution of DC between EZ and NEZ may due to brain parcellation with different atlas. Compared with the gross anatomy atlas, BA atlas parcellates brain into regions more detailly. Some high-amplitude CCEPs located in EZ might be assigned into the same BA region with other low-amplitude CCEPs of NEZ. Accordingly, the averaged responses of this region may get lower.

## Discussion

This study investigated the effective connectivity derived from direct electrophysiological recordings of CCEPs in eight medically intractable epilepsy patients, three different connectivity networks over different spatial scales were constructed for each patient. Graph theory was employed to analyze brain network topology, and graph metrics of EZ and NEZ were compared. We confirmed that connectivity networks reconstructed with CCEP amplitudes can indicate the effective connectivity of brain networks both at the electrode-pair-level and at the region-level. Importantly, EZ regions tend to have higher DC and NLP in comparison with NEZ, integration of local connectivity increased in regions of EZ.

### Effective Connectivity of Networks Reconstructed With CCEPs

In the last decades, direct cortical stimulation has been used as a useful investigational tool for epilepsy surgery, the evoked potentials CCEPs have been proved to be a powerful method for exploring the effective and functional connectivity in the living human. In our study, N1 potential of CCEP was regarded as the indication of the connection strength, which has been proved to be able to reflect the strength of connectivity between two brain regions (Fox et al., 2018). We fund that the effective networks observed from CCEP were asymmetric both in strength and in direction, CCEP connectivity networks mainly consisted with short-distance connections and few long-distance connections (see Figure 1, 2, 4). These findings are consistent with the results reported by Keller et al. (2014) who analyzed the brain network topology of 15 patients with medically intractable epilepsy. Trebaul et al. (2018) developed a large multicenter CCEP database with 213 epilepsy patients to analyze the human cortico-cortical connections. They also found that CCEP strengths were negatively corrected with the distance.

Furthermore, comparing with the other methods for CCEP quantitative analysis, like root mean square (RMS) (Enatsu et al., 2013) and analyzing the broadband gamma signals of CCEPs (Crowther et al., 2019), the way we used to quantify CCEPs is much easier and faster especially for the calculation of large sample size, and the important characteristics of CCEPs were preserved well. Additionally, in the constructed networks of our results, most of the regions located in PoG (postcentral gyrus), PrG (precentral gyrus) and frontal cortex exhibited strengthened connections, which is consistent with the distribution of hubs in the human brain network (van den Heuvel and Sporns, 2013). Entz et al. (2014) analyzed CCEPs from 25 refractory epilepsy patients and identified several major hub regions in the human brain, which mostly overlapped with the classical distribution of hubs. Together, these findings suggest that reconstructing effective brain networks with CCEP amplitudes we used is credible.

### Graph Metrics of Epileptogenic Zone

It has been recognized that epilepsy is a network disease of varying scales across multiple brain regions (Bartolomei et al., 2017). Moreover, the abnormal connectivity of brain networks has been proved to be associated with the localization of EZ, which may be a potential biomarker for the diagnosis and treatment of epilepsy. In this study, we applied two different brain atlases to parcellate brain areas and constructed brain networks with CCEPs at region level and found that alterations of effective network connectivity kept in line with the distributions of EZ. The connectivity matrices reconstructed with different atlases were similar to each other. Strong connections were observed among regions of EZ that exhibited higher effective connectivity than regions in NEZ (see in Figure 2, 4). The current findings are consistent with CCEP studies by Mouthaan et al. (2016) and Lagarde et al. (2018).

Moreover, many other researchers also have reported the high integration of effective connectivity and strong interictal connectivity of epileptogenic and propagation zones in epilepsy patients with EEG, MRI and fMRI. Tousseyn et al. (2017) used CCEPs and interictal single photon emission computed tomography (SPECT) to analyze network connectivity in 31 refractory focal epilepsy patients. They suggested that the distributions of hyper-perfusion in SPECT overlapped with the effective connectivity networks. This study combined functional connectivity and effective connectivity of the brain network, thus reconfirmed the reliability of CCEPs. Parker et al. (2018) found a significant overlap between structural networks of DTI and effective networks of CCEPs, and suggested structural connection strength in the epileptic focus tended to be higher. Hong et al. applied graph theory to analyze the structural connectivity and resting-state functional connectivity of 154 epilepsy patients and 82 healthy controls (Hong et al., 2017). Increased graph metrics were observed in EZ in the structural networks. Contrarily, inter-regional functional connectivity was decreased in regions of EZ because of the formal structure-function coupling. Overall, our findings are supportive to the concept of hyperexcitable cortex of EZ (Valentín et al., 2005; Bartolomei et al., 2017). That is, there is an imbalance between excitation and inhibition of activities in EZ, and the cortex excitability of focus areas is higher than others. Despite of the multifactorial nature of epilepsy and its heterogeneity, our study analyzed the effectivity network connectivity of CCEPs at region level and revealed the group pattern of network abnormalities of EZ.

Notably, the connectivity of networks constructed with different atlases was not exactly the same. For example, in the network of patient P1 that was constructed with the gross anatomy atlas, regions of EZ, inferior temporal gyrus (ITG) and middle temporal gyrus (MTG), only connected with each other, which can be seen in Figure 2A. As shown in Figure 3A, strong connections were also observed in the corresponding EZ regions BA21 and BA22 in the effective network based on BA atlas of P1. But region BA22 also connected with BA44, which located in the frontal gyrus. When comparing the networks with different spatial scales, the differences of graph measures between EZ and NEZ in networks based on the gross anatomy atlas seemed to be more significant in comparison with the ones computed from networks based on the BA atlas. This may be due to the inappropriate assignations of electrodes when we constructed brain networks at region level, significant CCEP responses of EZ may be averaged with the insignificant CCEPs of NEZ mistakenly. Also, epileptogenic cerebral lesion not respect for anatomic boundaries and the inappropriate electrode localization also had an impact on the effective connectivity of brain networks.

However, there are some limitations in this presented study. Only 8 epileptic patients with multiple anatomical locations were included. On one hand, the limited number of epilepsy patients and the different anatomical EZ locations of these patients could have reduced the statistical power of the data. On the other hand, the small spatial sampling CCEP signals available in a single patient could have made the study of effective connectivity in a limited scale, and the connectivity estimated from CCEP amplitudes depends on the stimulation parameters partially. More patients with the same anatomical locations of EZ and smaller individual differences will be included in our further study. Furthermore, in our results, the network reconstructed with the gross anatomy atlas seemed to perform better in the localization of EZ than the network based on BA atlas. More samples are needed to verify this result, and many other brain atlases should also be included in further studies. In addition, as recording ECoGs with electrode grids implanted on the brain cortex is invasive, it is impossible to compare the difference of effective connectivity networks between epileptics and healthy controls. Other measures of effective connectivity, like DTI, MRI and high-density EEG recordings can be used for comparison, combined with CCEP in the following study.

## Conclusion

We proposed a batch processing application of CCEPs based on MATLAB, and described the graph theory we used to analyze the topology of brain networks derived from CCEPs. We also explored the localization of the EZ with graph metrics of effective network. CCEPs recorded from patients with medically refractory epilepsy reflected the asymmetric distribution of brain network connectivity. Brain networks mainly consisted of short-distance connections. Regions in the EZ usually had higher DC and NLP than those out of the zone. This information has great potential to be applied to localize the epileptic focus clinically.

In summary, the analysis of complex brain network connectivity based on the feature extraction of CCEPs can provide effective and accurate information relating to the localization and delineation of EZ, thus helping epileptologists to make appropriate clinical decisions.

## Ethics Statement

This study was approved by the Medical Research Ethics Committee at Xuan Wu Hospital of Capital Medical University and written informed consents were obtained from all patients.

## Author Contributions

CZ and YL designed this study, implemented the algorithms, performed the data analysis, and wrote the manuscript. CL, RG, and JW selected and pre-processed EEG data. RZ, YZ, ZR, and XG performed the data analysis and helped to produce tables and figures. GZ and XZ provided the research ideas and revised the manuscript. All authors revised and approved the final version of the manuscript.

## Conflict of Interest Statement

The authors declare that the research was conducted in the absence of any commercial or financial relationships that could be construed as a potential conflict of interest.
